# Infants' Food

**Published:** 1891-01-10

**Authors:** 


### INFANTS' "FOODS."

An artificial food for infants which in the relative proportions of its constituents, and in the properties of the several carbo-hydrates, shall correspond to human milk, or which, when added to milk and water, shall not materially alter the resultant proportions, is still to be found. Prof. Escherich has published the results of a complete analysis of a number of "foods," from which we will take only those of two, Nestlé's and Neave's, as the others are not known in this country. The enormous difference in the percentages exhibited by these shows that, whichever be the better in one respect or another, both cannot be in the right.

| Food. | Water. | Albumenates. | Fat. | Grape Sugar. | Cane Sugar. | Starch and Dextrin. | Total Carbohydrates. | Proportion of Albmn. to Carbohydrates. | Ash. |
|---|---|---|---|---|---|---|---|---|---|
| Nestlé's | 6·3 | 8·39 | 5·31 | 4·61 | 33·21 | 38·95 | 76·77 | 1·9 | 2·05 |
| Neave's | 3·0 | 12·1 | 2·6 | 2·17 | traces | 69·03 | 71·20 | 1·56 | 3·2 |

Percentage to the Total Dry Substances of the Starch and Dextrin.

| | Soluble in Water. | Dissolved by Diastase. | Dissolved by Steam. | Total Carbohydrates deducting Sugar. |
|---|---|---|---|---|
| Nestlé's | 16·8 | 19·68 | 24·57 | 38·95 |
| Neave's | 13·11 | 16·24 | 39·68 | 69·03 |

Percentage of Sugar to the total Carbohydrates.

